# Technique of ICP Monitored Stepwise Intracranial Decompression Effectively Reduces Postoperative Complications of Severe Bifrontal Contusion

**DOI:** 10.3389/fneur.2016.00056

**Published:** 2016-04-11

**Authors:** Guan Sun, Lei Shi, Tianhong Pan, Xiaoliang Li, Shuguang Zhang

**Affiliations:** ^1^Department of Neurosurgery, Fourth Affiliated Yancheng Hospital of Nantong University, Yancheng, China; ^2^Department of Neurosurgery, The First People’s Hospital of Kunshan affiliated with Jiangsu University, Suzhou, China

**Keywords:** intracranial pressure, stepwise, intracranial decompression, bifrontal contusion, perioperative complications

## Abstract

**Background:**

Bifrontal contusion is a common clinical brain injury. In the early stage, it is often mild, but it progresses rapidly and frequently worsens suddenly. This condition can become life threatening and therefore requires surgery. Conventional decompression craniectomy is the commonly used treatment method. In this study, the effect of intracranial pressure (ICP) monitored stepwise intracranial decompression surgery on the prognosis of patients with acute severe bifrontal contusion was investigated.

**Method:**

A total of 136 patients with severe bifrontal contusion combined with deteriorated intracranial hypertension admitted from March 2001 to March 2014 in our hospital were selected and randomly divided into two groups, i.e., a conventional decompression group and an ICP monitored stepwise intracranial decompression group (68 patients each), to conduct a retrospective study. The incidence rates of acute intraoperative encephalocele, delayed hematomas, and postoperative cerebral infarctions and the Glasgow outcome scores (GOSs) 6 months after the surgery were compared between the two groups.

**Results:**

(1) The incidence rates of acute encephalocele and contralateral delayed epidural hematoma in the stepwise decompression surgery group were significantly lower than those in the conventional decompression group; the differences were statistically significant (*P* < 0.05); (2) 6 months after the surgery, the incidence of vegetative state and mortality in the stepwise decompression group were significantly lower than those in the conventional decompression group (*P* < 0.05); the rate of favorable prognosis in the stepwise decompression group was also significantly higher than that in the conventional decompression group (*P* < 0.05).

**Conclusion:**

The ICP monitored stepwise intracranial decompression technique reduced the perioperative complications of traumatic brain injury through the gradual release of ICP and was beneficial to the prognosis of severe traumatic brain injury treatment.

## Introduction

Bifrontal contusion is clinically common and mainly caused by direct impact on the forehead or either accelerated or decelerated force exerted on the occipital region (1). Bifrontal contusion can cause emergency central herniation, leading to severe disability or death. Central herniation is also known as bilateral tentorial herniation and often occurs as either bilateral space-occupying lesions (acute brain injury, cerebrovascular disease, or diffuse cerebral edema) or spontaneous intracranial hypotension caused during lumbar drainage, which shifts the brain hemisphere and basal ganglia downward through an oppression at the tentorial notch.

**Figure 1:**
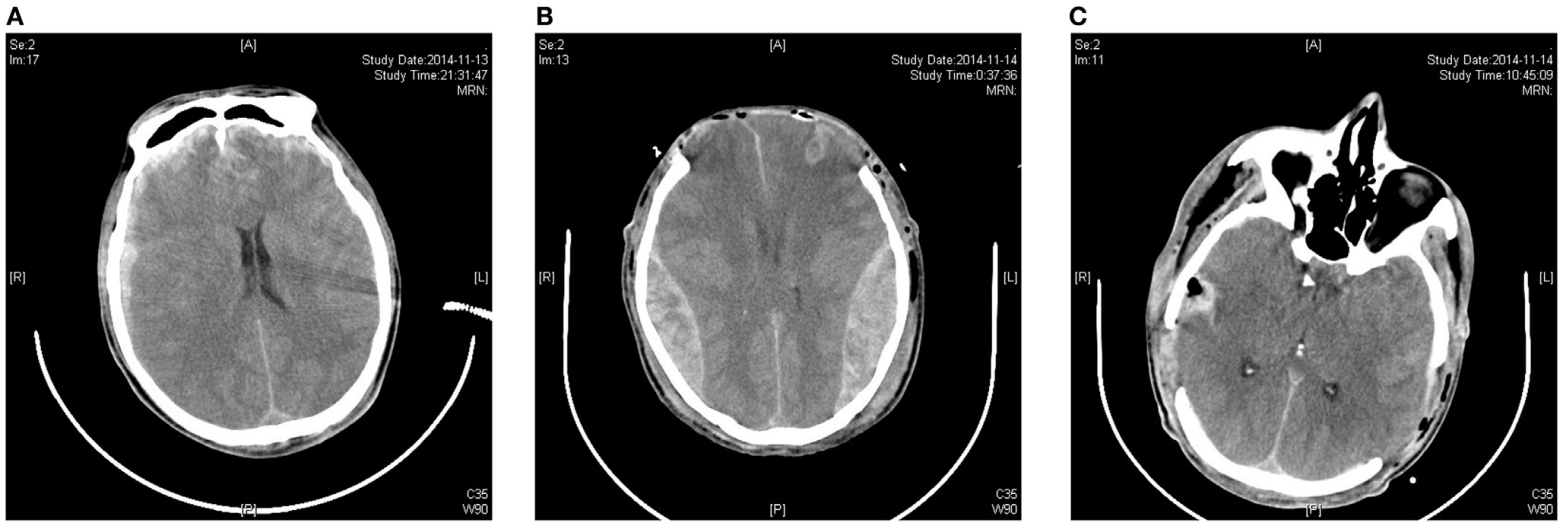
**A rapid decompression-induced acute encephalocele further induces the occurrence of the contralateral epidural hematoma**. **(A)** Preoperative CT skull hints double frontal cerebral contusion; **(B)** postoperative CT skull hints the bilateral temporal occipital epidural hematoma; **(C)** postoperative CT skull hints bilateral epidural hematomas completely cleared.

Currently, based on the clinical manifestations of central brain herniation or the diencephalon period, and combined with imaging features, it is believed that decompression craniectomy should be performed as early as possible to reduce the mortality and disability rate derived from central herniations caused by bifrontal contusions and to improve the cure rate of bifrontal contusion. However, acute encephalocele is a critical situation in a conventional severe bifrontal decompression craniectomy (2); once this condition occurs, it is catastrophic for the patient, and the surgeon also bears an enormous psychological burden. Also a rapid decompression induced acute encephalocele could further induce the occurrence of the contralateral epidural hematoma, which was shown in Figure 1.

Through retrospective study, we found that the ICP monitored stepwise intracranial decompression technique can effectively treat intraoperative acute encephalocele and can reduce the incidence of the contralateral delayed epidural hematoma, which is vital to the improvement of prognosis of patients with severe bifrontal contusion.

## Materials and Methods

### General Information

A total of 136 patients with severe head injury admitted from March 2001 to March 2014 in our hospital were included in this study. There were 80 males and 56 females, with an age range of 23–55 years old and an average age of 37.6 ± 2.3 years old. The injuries were all caused by car accidents, and the occipital region was the force impact location. The patients were randomly divided into two groups: the ICP monitored stepwise intracranial decompression group and the conventional decompression group, each with 68 patients. There were no statistically significant differences in terms of sex ratio, age, preoperative Glasgow outcome score (GOS) between the two groups. The study was approved by the ethics committee of the hospital, and informed consent forms were signed by the family members of the patients.

### Emergency Surgical Indications

When admitted, the patients were conscious. However, 6–72 h after admission, patients were experiencing symptoms such as significant irritability, decreased consciousness, and reduced or enlarged pupils. Dynamic brain computed tomography (CT) scans indicated cerebral contusion and increased progressive cerebral edema; thus, patients were promptly subjected to the decompression craniectomy.

### Exclusion Criteria

(1) Primary brain stem injury; (2) complications in vital organs, such as the heart, liver, lungs, and kidneys; (3) complications involving hemorrhagic shock, severe coagulation abnormalities, and multiple organ failure; and (4) histories of brain tumors and cerebral infarction.

### Surgery and Treatment Methods

Routine preoperative preparations included dehydration and diuretic therapy. General anesthesia with endotracheal intubation was performed. Codman ICP Express intracranial pressure (ICP) monitoring equipment was also prepared.

#### Surgical Methods

##### Conventional Decompression Group

All patients received the bifrontal lobe double coronal approach and decompression craniotomy, in which the bone window was opened to the orbital rim, and the meninges were fully open to expose the frontal pole. Any bleeding, lifeless brain contusion tissues were cleared to conduct the intracranial decompression. Postoperative drainage tubes and intracranial monitoring pipes were placed.

##### Stepwise Decompression Group

In addition to the treatments employed in the conventional decompression group, one to two incisions of 0.5–1 cm in length were made in the bilateral dura. After opening the dura, a suction tip was instantly positioned at the epidural incision; if there was bloody cerebrospinal fluid outflow or hematoma, a small piece of cotton padding was then placed at the incision and held against the suction tip to slowly reduce ICP until no liquid drained out. Then ICP probe was implanted into the frontal lateral ventricle or the brain parenchyma. When the ICP was monitored, the dura at one side could be opened if the ICP had dropped to 20–25 mmHg or less, and the brain contusion and hematoma were further cleared. After complete hemostasis, compression hemostasis was performed with cotton padding. The dura was then opened on the other side, and the brain contusion and hematoma tissues were cleared after suspension. If the brain tissue pressure was still too high during the stepwise decompression process, leading to high dural tension, and the ICP was over 25 mmHg, the dura should be cut open with mesh-style cuttings while it was rapidly infused with intravenous drips of mannitol and furosemide and hyperventilation. The brain contusion and hematoma could then be cleaned through the mesh-style openings of the dura; the clearing was performed similarly on both sides, as mentioned previously. If the ICP was below 25 mmHg during the cleaning, the dura was fully opened again to clear out the brain contusion and hematoma. However, if the ICP did not decrease, or if it increased, and the brain tissue began to squeeze out of the mesh-style dural opening after performing the above-mentioned stepwise decompression method, the brain contusion tissue and subdural hematoma were removed from the dural opening as much as possible according to the patient’s intraoperative vital signs; the dura should not be fully opened (3).

### Observation Items

Changes in ICP in both groups: the incidence rates of acute brain swelling and delayed brain hematoma; the incidence rate of postoperative cerebral infarction; and GOS assessment of prognosis 6 months after treatment.

### Statistical Methods

SPSS 16.0 software was used in the analysis. For data measurements, means ± SD were used. Intergroup comparisons of the measured data were performed using the independent samples *t* test. The chi-square test was used to compare the enumeration data. *P* < 0.05 was considered statistically significant.

## Results

### Stepwise Decompression Surgery Significantly Reduced the Incidence of Intraoperative Acute Encephalocele

This retrospective study found that among the 68 patients who received the stepwise decompression surgery, only 3 patients developed intraoperative sustained acute encephalocele, with an incidence rate of 4.4%; these 3 cases all had poor prognosis, 2 of which died after active intensive care unit (ICU) rescue, and 1 entered a vegetative state. Among the 68 patients who received the conventional decompression surgery, 17 patients developed intraoperative sustained acute encephalocele, with an incidence rate of 25%. These 17 cases had poor prognosis; 11 died after active ICU rescue, and the remaining 3 cases entered a vegetative state. When the two groups were compared, the difference was significant, indicating that the stepwise decompression surgery significantly reduced the incidence of acute encephalocele in patients with severe bifrontal contusions.

### Stepwise Decompression Surgery Significantly Reduced the Incidence of Contralateral Epidural Hematoma in the Early Postoperative Period

In conventional intracranial decompression surgery, after the rapid removal of intracranial hematoma, the dura was detached from the skull internal lamina, or bridging veins were torn off to form a contralateral epidural hematoma; the filling pressure effect then induced fast shifting of the intracranial contents. Among the 68 patients who received the stepwise decompression surgery, no contralateral epidural hematomas occurred. However, in the 68 patients who received the conventional decompression surgery, 7 patients developed sustained intraoperative contralateral epidural hematoma, with an incidence rate of 10.3%. Among these patients, 2 had bilateral epidural hematoma, and 5 had unilateral epidural hematoma; 3 of the 7 patients underwent emergency surgery, and the remaining 4 cases underwent conservative therapy. These results demonstrated that, compared with conventional decompression surgery, the stepwise decompression surgery significantly reduced the incidence of contralateral epidural hematoma during the decompression process in patients with severe bifrontal contusion.

### Stepwise Decompression Surgery Significantly Reduced the Incidence of Vegetative State and Mortality in the Late Postoperative Period

Intraoperative acute encephalocele during surgeries for severe brain injury has been a critical issue in neurosurgery, leading to high mortality and morbidity. This retrospective study found that stepwise decompression surgery reduced the incidence of acute encephalocele; patient morbidity and mortality were also significantly decreased. Among the 68 patients who received the stepwise decompression surgery, only 2 patients died, with a mortality of 2.9%; and 3 patients entered a vegetative state, with an incidence rate of 4.4%. Among the 68 patients who received the conventional decompression surgery, 11 died, with a mortality of 16.2%; 7 entered a vegetative state, with an incidence rate of 10.3%.

## Discussion

Bifrontal lobe injury is usually caused by contrecoup injury resulting from occipital impact, leading to severe frontotemporal cerebral edema or intracerebral hematoma formation, which sharply increases the ICP, compresses brain tissue in the frontal pole direction, and exerts pressure on the diencephalon and brain stem to form a central herniation (4). Contrecoup bifrontal contusion can easily lead to central herniation, which has a bottom-up development process in which the diencephalon is first affected; the contusion then sequentially expands to the mesencephalon, pontine, and medulla, i.e., bilateral transtentorial herniation occurs, and the brainstem shifts downward along the central axis, which leads to compression, stretching, and ischemia in the aforementioned brainstem structures (2). In severe cases, bifrontal contusion can lead to compression on the respiratory and cardiovascular centers in the reticular formation and can ultimately lead to irreversible coma or death (5). A central herniation progress rapidly, has a short course, and may cause sudden respiratory and cardiac arrest in patients (1). Sekula and other types of central herniations do not cause alterations to the pupils in the early period. The pupil usually narrows initially, and then rapidly dilates over a short period of time, and the light reflex disappears. A brain CT will show extensive bifrontal contusion with severe brain swelling and bilateral cerebral ventricle compression on the frontal eminences, along with obvious compression on the cisterna, including the ambient cistern, the cistern, and the quadrigeminal cistern. The midline usually does not shift remarkably; thus, midline shift is separated from the illness. Magnetic resonance imaging (MRI) can often display the typical symptoms: the midline structure on the tentorial edge shifts downward, and the swollen midbrain also shifts downward below the tentorial, accompanied by severe brain swelling. The basal cistern, especially the suprasellar cistern and other regions around the midbrain, are blocked, and the diencephalon is pressed toward the saddle back. The tectum of the mesencephalon shifts downward and backward, while the fastigium is pressed backward, and the bilateral red nucleus significantly shifts downward below the tentorial.

Although the brain injuries induced by bifrontal contusion can be asymmetrical, most scholars have believed that simultaneous bilateral craniectomy for balanced decompression should be conducted (6). In such cases, at least two neurosurgeons with certain experience in traumatic brain injury rescue are required to perform the craniotomy. The flap and bone graft designs should be selected according to the severity of the brain damage, using either a coronary approach or a parafontalia on one side and a pterion on the other side; the bone graft should be lowered to the level of the orbital rim, and the bifrontal contusion necrosis and intracerebral hematoma should be removed and cleared. For patients with pupil changes, bilateral decompression craniectomy is preferred.

The key issue is how to reduce the acute encephalocele and delayed formation of hematoma in patients who receive rapid decompression surgery.

At present, it is thought that the possible mechanism of acute intraoperative encephalocele is that, as the dura is suddenly opened or the hematoma suddenly removed, there is a sharp decline in ICP, and the cerebral vasculature dilates passively, leading to brain hyperemia and swelling, resulting in brain tissue continuously swelling toward the bone window and causing incarcerated congestion in the vessels on the brain surface at the edge of the bone window and disorders in cerebral venous drainage, further aggravating the brain swelling and resulting in a vicious cycle (6). Eventually, large masses of swollen tissue burst out of the bone window, rendering it impossible to close the incision, and the swollen brain tissue has to be incised to forcibly close the cranial opening, leading to a very low survival rate in affected patients (7). The occurrence of delayed intraoperative hematoma mainly consists of epidural hematoma at the impact site (8). There are a couple of possible reasons for its occurrence (9). First, a sudden decrease in ICP leads to the disappearance of the pressure tamponade effect (10); because preoperative intracranial hypertension has a hemostatic effect, after craniectomy, the outer subdura is cut open, the hematoma is cleared, the hemostatic effect disappears or decreases, and the bleeding rapidly forms a hematoma or increases an existing hematoma (11). Second, surgical decompression causes the rapid displacement of cranial contents, leading to the detachment of the dura from the skull internal lamina or tearing off of the bridging veins and, ultimately, the formation of a hematoma (12).

In the present study, an ICP monitored stepwise intracranial decompression technique was used in which a gradual reduction in ICP was adopted during the decompression craniotomy process, leading to the reduction in the formation of acute encephalocele and delayed hematoma induced by rapid decompression in the craniectomy process. The incidence rates of intraoperative acute encephalocele and delayed hematoma in the stepwise decompression craniectomy were significantly lower than those in the conventional decompression craniectomy, indicating that the stepwise decompression craniectomy could effectively reduce the intraoperative and early postoperative acute complications to avoid secondary brain damage. At the same time, the short-term (6-month) outcomes of the patients in the two groups were compared: 6 months after the surgery, the prognosis in the patients from the stepwise decompression group was significantly better than that in the patients from the conventional decompression group, and the incidence rates of vegetative state and mortality were significantly reduced, further verifying the clinical efficacy of the ICP monitored stepwise intracranial decompression technique. During the process of this method, it needs two neurosurgeons cooperation, who should be familiar with the operation. And an extra cost for this advanced technology is for each patient.

In summary, in this study, by employing the gradual release of ICP, the ICP monitored stepwise intracranial decompression technique could significantly reduce the intraoperative and postoperative complications in patients suffering from severe bifrontal brain injury. These findings have high significance in improving the treatment and prognosis of severe bifrontal brain injuries.

## Author Contributions

LS: participated in surgery and retrospective analysis, and writing this manuscript. GS, TP, and XL: participated in surgery. SZ: participated in surgery and retrospective analysis.

## Conflict of Interest Statement

All authors have declared the sources of research funding for this manuscript and have no financial or other contractual agreements that might cause (or be perceived as causes of) conflicts of interest.
